# Current practice and recommendations for advancing how human variability and susceptibility are considered in chemical risk assessment

**DOI:** 10.1186/s12940-022-00940-1

**Published:** 2023-01-12

**Authors:** Julia R. Varshavsky, Swati D. G. Rayasam, Jennifer B. Sass, Daniel A. Axelrad, Carl F. Cranor, Dale Hattis, Russ Hauser, Patricia D. Koman, Emily C. Marquez, Rachel Morello-Frosch, Catherine Oksas, Sharyle Patton, Joshua F. Robinson, Sheela Sathyanarayana, Peggy M. Shepard, Tracey J. Woodruff

**Affiliations:** 1 Department of Health Sciences and Department of Civil and Environmental Engineering Northeastern University, Boston, MA 02115 USA; 2 Department of Obstetrics, Program on Reproductive Health and the Environment, Gynecology and Reproductive Sciences, University of California, San Francisco, San Francisco, CA USA; 3 Natural Resources Defense Council, Washington, DC USA; 4 Independent consultant, Washington, DC USA; 5 Department of Philosophy, University of California, Riverside, Riverside, CA USA; 6 Environmental Toxicology Graduate Program, College of Natural and Agricultural Sciences, University of California, Riverside, Riverside, CA USA; 7 The George Perkins Marsh Institute, Clark University, Worcester, MA USA; 8 Department of Environmental Health, T.H. Chan School of Public Health, Harvard University, Boston, MA USA; 9 Department of Environmental Health Sciences, School of Public Health, University of Michigan, Ann Arbor, MI USA; 10 Pesticide Action Network, Berkeley, CA USA; 11 School of Public Health, University of California, Berkeley, Berkeley, CA USA; 12 Department of Environmental Science, Policy and Management, University of California, Berkeley, Berkeley, CA USA; 13 University of California, San Francisco School of Medicine, San Francisco, CA USA; 14 Commonweal, Bolinas, CA USA; 15 Center for Reproductive Sciences and Department of Obstetrics, Gynecology & Reproductive Sciences, University of California, San Francisco, San Francisco, CA USA; 16 Department of Pediatrics, University of Washington, Seattle, WA USA; 17 Seattle Children’s Research Institute, Seattle, WA USA; 18 WE ACT for Environmental Justice, New York, NY USA

**Keywords:** Adjustment factors, Chemicals, Cumulative risk, Environmental justice, EPA, NAMs, Risk assessment, Susceptibility, Variability, Vulnerability

## Abstract

A key element of risk assessment is accounting for the full range of variability in response to environmental exposures. Default dose-response methods typically assume a 10-fold difference in response to chemical exposures between average (healthy) and susceptible humans, despite evidence of wider variability. Experts and authoritative bodies support using advanced techniques to better account for human variability due to factors such as in utero or early life exposure and exposure to multiple environmental, social, and economic stressors.

This review describes: 1) sources of human variability and susceptibility in dose-response assessment, 2) existing US frameworks for addressing response variability in risk assessment; 3) key scientific inadequacies necessitating updated methods; 4) improved approaches and opportunities for better use of science; and 5) specific and quantitative recommendations to address evidence and policy needs.

Current default adjustment factors do not sufficiently capture human variability in dose-response and thus are inadequate to protect the entire population. Susceptible groups are not appropriately protected under current regulatory guidelines. Emerging tools and data sources that better account for human variability and susceptibility include probabilistic methods, genetically diverse in vivo and in vitro models, and the use of human data to capture underlying risk and/or assess combined effects from chemical and non-chemical stressors.

We recommend using updated methods and data to improve consideration of human variability and susceptibility in risk assessment, including the use of increased default human variability factors and separate adjustment factors for capturing age/life stage of development and exposure to multiple chemical and non-chemical stressors. Updated methods would result in greater transparency and protection for susceptible groups, including children, infants, people who are pregnant or nursing, people with disabilities, and those burdened by additional environmental exposures and/or social factors such as poverty and racism.

## Main text

A critical element affecting the accuracy and usefulness of chemical risk assessment is accounting for the full range of individual and population variability in response to environmental chemical exposures. There are four stages of risk assessment, including (1) hazard characterization, (2) exposure assessment, (3) dose-response modeling, and (4) risk characterization. Although it is important to characterize variability in exposure, dose-response, and risk, this paper focuses on the importance of characterizing variability in the dose-response relationship between a chemical and health outcome as a critical part of predicting population health risks for both cancer and non-cancer health risks. Issues regarding the underestimation of exposure variation are discussed in more detail by Vandenberg et al. in the special topics companion paper. Fully describing human variability is a necessary step towards ensuring the protection of everyone, including those most susceptible and highly exposed. Understanding those who are in the highest exposed group and/or who are most affected can help target interventions and policies that protect those most at-risk and subsequently the whole population. While many intrinsic and extrinsic factors, including age/life stage of development, genetics, underlying physiological function, and socioeconomic status (SES), can either separately or together enhance individual susceptibility and affect population variability in response to chemical exposures [1–4], current dose-response assessment methods often do not adequately account for these factors [5, 6]. For example, current methods often do not account for in utero susceptibility to chemical exposures, despite ample scientific literature demonstrating increased susceptibility among developing fetuses and the potential for fetal origins of disease [7–11].

Authoritative expert committees have recommended increased efforts to account for susceptible and highly exposed populations [1, 12–14]. The 2004 National Environmental Justice Advisory Council (NEJAC) report on cumulative risk assessment emphasized that incorporating the full range of stressors to which populations are exposed is key to understanding community risk and community health [14]. This is consistent with recommendations of the National Academy of Sciences (NAS) [1, 12, 13] and scientific articles [6, 15, 16] which conclude that default approaches to treatment of human variability in risk assessments need to be updated to better incorporate current knowledge regarding human variability and susceptibility factors.

Multiple United States (US) laws also require that there be adequate consideration of risks to susceptible populations and communities that are marginalized. The 2016 Frank Lautenberg Chemical Safety for the 21st Century Act (Lautenberg TSCA), which amended the 1976 Toxic Substances Control Act (TSCA), mandates protection of “potentially exposed and susceptible subpopulations” [5], and the Clean Air Act requires that National Ambient Air Quality Standards (NAAQS) be set with an “adequate margin of safety” to protect public health [2, 17]. Nevertheless, updates to methods for hazard, dose-response, and risk assessment to account for these legally required mandates have been limited [5, 6, 18], and thus risks are underestimated for susceptible subgroups, such as pregnant people [2], developing fetuses/neonates, children/adolescents, low SES communities, those with preexisting disease or lower physiological function, disabilities, genetic susceptibility, and those burdened by additional occupational and/or environmental exposures [1, 12, 13].

In this review, we describe the intrinsic and extrinsic factors that influence human variability and heighten susceptibility to toxic environmental exposures in human health risk assessment. We further describe current US approaches and existing frameworks for addressing variability in dose-response assessment. We then discuss key scientific inadequacies necessitating improved methods, as well as emerging tools and opportunities for improvement. We conclude with a discussion of specific and quantitative recommendations for implementing feasible changes to current practice that would ultimately advance human health risk assessment and ensure the protection of everyone, including those most susceptible and highly exposed. For this paper, we reviewed and collected evidence from authoritative bodies, such as the Environmental Protection Agency (EPA) and the NAS, as well literature searches based on common risk assessment terms.

### Sources of human variability and susceptibility in dose-response assessment

**Fig. 1:**
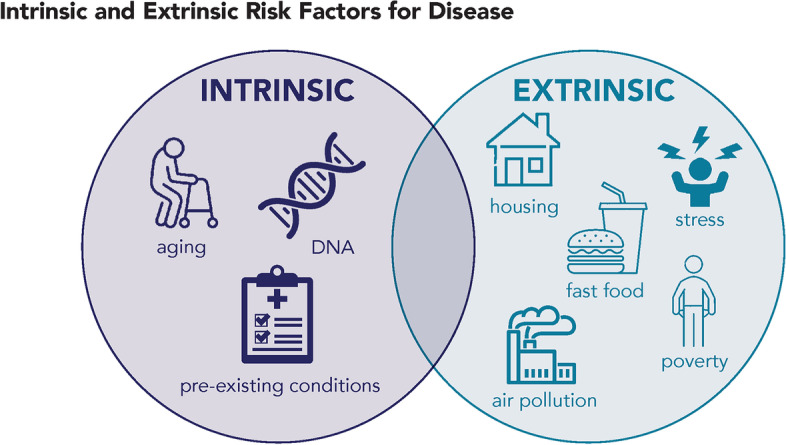
Sources of human variability and susceptibility to disease risk from exposure to environmental chemicals and pollutants

There are multiple factors that can influence disease risk and human variability in response to environmental exposures, some of which are intrinsic (or biological) host susceptibility factors, while others are extrinsic (or external) susceptibility factors that are potentially modifiable (Fig. 1). Variability refers to the variation in response to chemical exposures across the human population, which can be influenced by intrinsic and extrinsic risk factors, whereas susceptibility refers to the risk difference associated with these risk factors [19]. However, it is worth noting that not all intrinsic or extrinsic factors enhance variability or susceptibility.

Intrinsic factors that heighten individual susceptibility to environmental exposures include biological factors, such as genetic make-up or DNA, preexisting disease status or underlying health conditions, lower physiological function, and early life stage of development or aging [1] (Fig. 1). Age or life stage of development play a critical role in biological susceptibility because chemicals that are biologically active, such as endocrine disrupting chemicals (EDCs), can interfere with physiological processes at low levels of exposure that can influence human reproduction, development, and function [7]. For example, humans are more susceptible to endocrine-disrupting effects during critical stages of biological change and development, such as in utero, during puberty, and during pregnancy [11, 20]. Human variability in the absorption, distribution, metabolism, and elimination of chemicals can also influence disease risk, and age-dependent biological changes associated with various developmental stages can increase health risks associated with chemical exposures across the life course. For example, in utero changes are intrinsic susceptibility factors that are most relevant for fetal growth and development as well as prenatal programming of future health risks and susceptibility to chemical exposures [1, 21]. Other notable developmental stages include early childhood and adolescence, which are susceptible times of hormone-regulated growth that include the onset of puberty [21, 22]; pregnancy, a period of rapid biological changes that can impact lifelong health risks [20]; and the physiological changes associated with aging which can also increase health risks associated with chemical exposures [23, 24].

Extrinsic factors, such as nutritional status and exposure to multiple chemicals or pollutants (through additional exposure pathways, for example, from diet, housing or the built environment, and ambient air pollution) and/or non-chemical stressors (including psychosocial stressors and social or economic factors such as poverty) [1, 6], can also enhance susceptibility to environmental chemical exposures [25] (Fig. 1). Current risk assessment practice does not typically factor in these enhanced risks or seek to quantify interactions. Since these external risk factors affect communities that have been historically marginalized, ensuring that risk assessments capture extra susceptibilities is important for environmental justice. Communities of color, low-wealth communities, and Indigenous communities face greater exposure to environmental and health hazards compared to communities with more white or affluent people. For example, these communities are burdened by disproportionate numbers of toxic “legacy” sites (e.g., former industrial areas, military facilities, closed or abandoned contaminated sites) [26, 27]; large numbers and concentrations of chemical storage and industrial facilities that release toxic substances into the air, water, or soil and may present an ongoing risk or potentially a chemical disaster [28, 29]; increased rates of drinking water violations [30]; air pollution from heavy traffic or ports [26, 29, 31–34]; and, exposure to toxic chemicals and other contamination in consumer products, pesticides, food, and air [35–38]. These toxic exposures are often patterned on racial segregation and discrimination, economic inequities, and political barriers [18, 27, 31, 33, 34, 39, 40], with these exposure disparities further discussed in the companion paper on exposure by Vandenberg et al. in this issue. They can result in physiological changes (for example, changes to the immune, neuroendocrine, and cardiovascular systems) [41], and are linked to social disadvantage and deleterious health outcomes including lower life expectancy [42], higher preterm birth rates [43], low birthweight [18, 42, 44–46], cardiovascular disease and hypertension [47, 48], autoimmune diseases [49–55], asthma [56, 57], diabetes [58, 59], cancer [60, 61], and infectious diseases [62, 63]. Thus, improving regulatory risk assessments to sufficiently account for the full extent of human variability is a critical step towards improved environmental conditions and health in communities that have been disproportionately exposed and marginalized.

Extrinsic and intrinsic factors can interact to enhance health risks among susceptible populations. Life stage and health status (for example, infancy, pregnancy, older age, underlying health conditions) can heighten biological sensitivity [11, 64], while socially patterned factors (e.g., poverty, racism) can also deprive groups of access to mitigating factors in a systematic manner (e.g., access to health care) [65–67]. These multiple health, social, and environmental hazards create interconnected, cumulative impacts in historically and currently disadvantaged populations, which adversely impact health and thus limit the ability to grow and thrive [68, 69], and which are not adequately accounted for in standard default risk assessment methods.

**Fig. 2:**
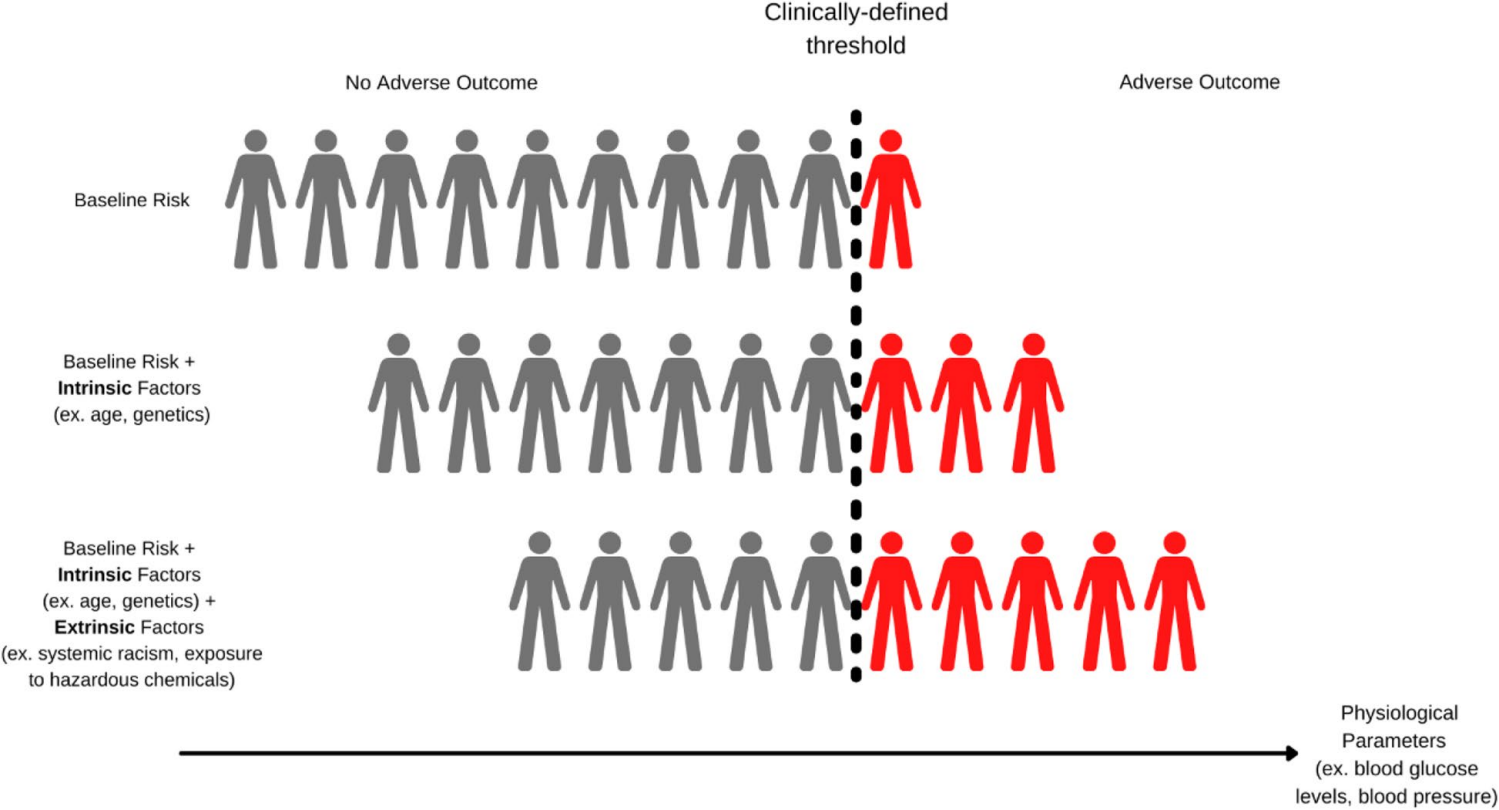
How intrinsic and extrinsic factors can influence risk of an adverse outcome In this illustration, a physiological parameter value greater than the clinically-defined threshold results in an adverse outcome or diagnosed disease in the general population (baseline risk). With intrinsic factors alone, some additional proportion of the population may have a parameter value above the clinically-defined threshold and experience an adverse outcome (baseline risk + intrinsic factors). With the addition of extrinsic factors, such as exposure to hazardous chemicals and/or non-chemical stressors, values of the physiological parameter in the population increase, resulting in an increased proportion of the population above the clinically-defined threshold and thus experiencing the adverse outcome

These intrinsic and extrinsic factors can also either separately or together shift the human population (or susceptible subpopulations) of a physiological parameter in the direction of illness or disease (Fig. 2). For example, pregnancy is a critical period of maternal health that is accompanied by extreme changes to maternal physiology to accommodate the developing fetus. These changes can increase biological susceptibility to gestational diabetes by increasing maternal insulin resistance and moving pregnant people into a prediabetic borderline disease state, closer to the clinically-defined adverse health outcome threshold, which increases risk from subsequent chemical exposures [20]. Chemicals (such as metabolic disruptors) can further influence disease risk by shifting the population closer to the level of function indicating an adverse effect (e.g., by disrupting maternal insulin production in the pancreas) [20]. This heightened susceptibility combined with additional non-chemical co-exposures, such as psychosocial stress or economic/food insecurity, can further shift the risk distribution in the direction of clinical disease, effectively increasing the percent of the population with the clinical or apical outcome, which is explored in more detail by Nielsen et al. in the companion paper on probabilistic methods for estimating non-cancer risks and in the summary manuscript within this special topic series. Thus, the NAS concluded that while many individuals in the population may not have an observable outcome, the overall effect of a chemical exposure combined with other risk factors which vary across the population is that there is no expected threshold among the population for health risks from the chemical exposure [1]. Indeed, studies have shown these factors can interact to increase susceptibility to chemical exposures [1, 6]. Several have demonstrated additive and/or synergistic effects from exposure to multiple chemicals (e.g., anti-androgenic phthalates, thyroid-disrupting pesticides) [13, 70] and other (both intrinsic and extrinsic) factors (e.g., genetic predisposition, age/life stage, occupational exposure, poverty & malnutrition), which can increase risk among some susceptible subgroups.

### Existing US frameworks for addressing response variability in risk assessment

Characterizing the dose-response relationship between a chemical and health outcome is a critical part of predicting population health risks for both cancer and non-cancer health risks [1]. We begin by describing approaches to dose-response assessment for both cancer and non-cancer endpoints, then describing customary approaches to treatment of human variability in non-cancer risk assessments, including the default adjustment factor, as well as chemical-specific adjustment factor (CSAF) or data-derived extrapolation factor (DDEF), approaches.

#### Characterization of the dose-response relationship for cancer endpoints

Cancer endpoints have typically been characterized by a linear dose-response relationship, based on the assumption that any exposure level of the chemical is associated with some cancer risk that linearly increases with increasing exposure level–the slope of the line describes the potency or strength of the cancer risk [71, 72]. These linear dose-response curves facilitate extrapolation of cancer risk across a distribution of exposures, including at lower exposures that may be below the observable range of data. However, the dose-response for cancer is typically based on studies conducted in mature laboratory animals or human adults and otherwise lacks variability [1].

#### Characterization of the dose-response relationship for non-cancer endpoints

**Fig. 3:**
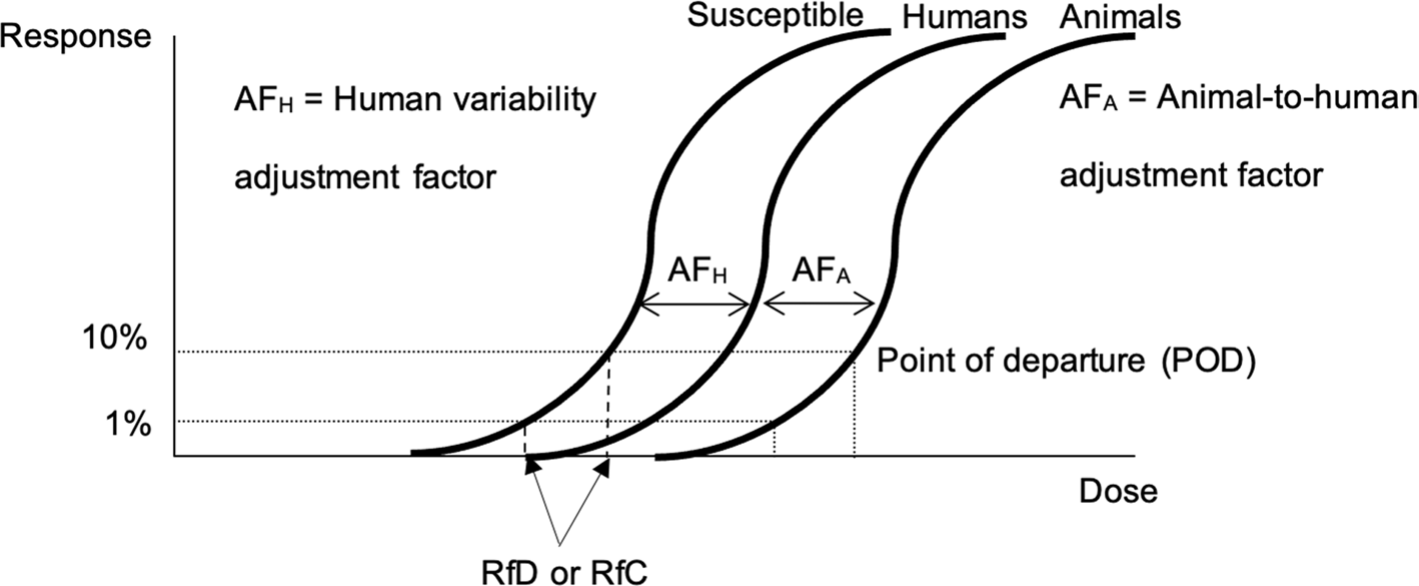
Depiction of US construct for deriving oral reference dose (RfD) and inhalation reference concentration (RfC) RfD and RfC defined as exposure estimates that are “likely to be without an appreciable risk of deleterious effects during a lifetime” for non-cancer endpoints in the human population (including susceptible subgroups), when using experimental animal data. The RfD or RfC is derived from an experimental animal point of departure (POD), such as the statistical lower limit on a benchmark dose (BMDL) that is associated with a pre-determined change in response. Adjustment factors (AFs) to account for inter-species (experimental animal-to-human) differences and intra-species (healthy humans to susceptible subgroups) variability are then applied to the experimental animal POD. The POD is divided by the animal-to-human AF (AFA) to extrapolate from animals to humans and by the human variability AF (AFH) to account for within-human population variability [73]

In contrast, the current chemical risk assessment approach by the US EPA and other federal agencies to evaluate non-cancer endpoints, such as reproductive and developmental problems, typically involves determining a reference value, e.g., the oral reference dose (RfD) or the inhalation reference concentration (RfC), which are defined by the US EPA as estimates of “daily oral exposure and continuous inhalation exposure, respectively, to the human population (including susceptible subgroups) that are likely to be without appreciable lifetime risk of deleterious effects” [71]. The RfD and RfC are typically used as ‘bright line’ values in which it is assumed that exposure above the RfD or RfC poses some unspecified degree of risk (not a probability of risk like in cancer risk assessment) and exposure below it poses zero risk. Both the RfD and RfC are typically derived from a point of departure (POD) representing the low end of the observable exposure-response relationship (e.g., 1% or 10%), with adjustment factors (AFs) generally applied to reflect data limitations and the inherent variation in susceptibility to chemical exposures between experimental animals and humans (inter-species variability) and among humans (intra-species variability) [71] (Fig. 3).

The traditional RfD/RfD does not provide a quantitative estimate of risk because it is defined in non-quantitative terms and has no scale which relates back to the probability of adverse outcome [71]. The assumption of a non-linear dose-response relationship has historically been used to justify no extrapolation of dose-response relationships to lower doses for non-cancer outcomes, due to uncertainty about the shape of the dose-response curve at doses below the observable range of data. Thus, the prevailing characterization of non-cancer risk has been a threshold effect [1, 71]. However, more contemporary science indicates that non-cancer endpoints (e.g., infertility, pregnancy complications, birth defects, neurodevelopmental delays, metabolic disorders, and cardiovascular disease) should be assumed to have non-threshold dose response relationships due to population variability in response [1, 6, 72]. Probabilistic methods for dose-response assessment of non-cancer effects, in place of current reference value approaches that establish a threshold, are addressed in more detail by the Nielsen et al. companion paper within this special issue.

#### Default adjustment factor approach

In the traditional RfD/RfC approach, inter- and intra-species variability are captured or represented by default uncertainty or AFs which are assumed to be default point estimates of 10X each for inter- and intra-species variability (Table 1). These 10X factors account for the default assumption that humans are more susceptible than animals by up to a factor of 10 (inter-species variability), and that susceptible humans are as much as 10 times more susceptible than average humans (intra-species variability). This 10-fold default adjustment factor was originated by Lehman and Fitzhugh, who in 1954 recommended deriving an acceptable margin of safety for humans using the maximum dose at which no effects were observed in animal experiments and dividing by 100 [74]. The 100X default factor was rationalized as a product of two 10-fold assessment factors, the first accounting for inter-species differences and the second for human variability. The resultant equation is thus: *RfD (or RfC) = POD / (AF*_A_* AF_*H*_) = POD / (10 * 10) = POD / 100. In the RfD/RfC approach, additional adjustment factors may be applied as appropriate for data limitations such as use of a subchronic study, use of a lowest observed adverse effect level (LOAEL) as POD, or an incomplete database. For example, while reference values derived from animal studies typically apply an adjustment factor of 10X for interspecies differences and an adjustment factor of 10X for human variation, a database uncertainty factor of 3X or 10X may also be applied when studies of important endpoints, such as developmental effects, are missing [71].

**Table 1:** US approaches to account for human variability in derivation of reference values for risk assessment

Approach	Intended Use	Method
Default adjustment factor (AF)	Adjust for total intra-species variability.	AF_H_ = 10.
Default subfactor approach	Adjust for intra-species variability in metabolism (TK) and tissue response (TD).	AF_H_ ≤ 10 (usually 3X each for AF_H-TK_ and AF_H-TD_).
Chemical-specific adjustment factor (CSAF) or data-derived extrapolation factor (DDEF)	Adjust for chemical-specific intra-species differences in metabolism (TK) and tissue response (TD).	AF_H-TK_ and AF_H-TD_ based on case-by-case TK/TD data.

*AF*_*H*_ Intra-species human variability adjustment factor, *AF*_*H-TK*_ Intra-species human variability adjustment subfactor for variation in kinetic chemical metabolism, *AF*_*H-TD*_ Intra-species human variability adjustment subfactor for variation in target tissue respons﻿e

#### Default adjustment subfactor approach

The default 10-fold adjustment factors for inter- and intra-species variability are often divided into two subfactors ranging from 2-fold to 10-fold (e.g., ≤10 = 3.16 rounded down to 3X) in order to represent the assumed different contributions of toxicokinetic (TK) and toxicodynamic (TD) variability to the combined AFs [16] (Table 1). The AF_H-TK_ (toxicokinetic variation) is meant to account for intra-species differences in human absorption, distribution, metabolism, and elimination of toxic chemicals (i.e., the way the body processes the chemical) while the AF_H-TD_ (toxicodynamic variation) is meant to account for response differences independent of the dose level at the target organ (i.e., the way the body responds to a given internal dose of the chemical at the target tissue), which can vary across the population by genetics, underlying physiological function, illness, age, and other factors.

#### Chemical-specific or data-derived approaches

CSAF or DDEF approaches allow risk assessors to deviate from using default adjustment values when chemical-specific TD/TK data are available which support different values for human variability [73, 75] (Table 1). These approaches encourage the integration of TK/TD data for individual chemicals to determine AF_H_ on a case-by-case basis. Importantly, there is overlap in the biological processes that contribute to TK and TD variability. For example, environmental exposures can alter tissues and hormones (i.e., toxicodynamics) that in turn change the distributions (or toxicokinetics) of chemical metabolism. Therefore, the split between TK and TD variability is somewhat arbitrary.

### Key scientific inadequacies necessitating improved methods

While adjustments to risk assessment practice have been made over time, each of the above risk assessment methods (summarized in Table 1) have limitations and commonly do not incorporate available data demonstrating a wider range of human variability in response to chemical exposures. While each approach is based on Toxicokinetic (TK)/Toxicodynamic (TD) data (biological variability), none of the current methods explicitly account for data demonstrating increased biological susceptibility and population variability due to social factors. Additionally, CSAF or DDEF approaches are highly dependent on robust data for individual chemicals assessed in order to develop appropriate chemical-specific factors [73, 75], and chemical-specific data are available in only rare cases.

Established and growing scientific evidence indicates that default adjustment factors of 10X or less are not adequate for protecting human health from chemical exposures. The NAS presented several examples of the inadequacy of the approach in the 2009 *Science and Decisions* report, showing how factors like genetic susceptibility, preexisting disease, and interacting chemical exposures can increase susceptibility by more than 10-fold compared to controls (Table 2). For example, smokers have been shown to be 20 times more susceptible to arsenic-induced lung cancer than non-smokers [76], and women who smoke and have low iodide levels were up to 100 times more susceptible to perchlorate-induced thyroid hormone disruption than their control counterparts [77]. Increased susceptibility to copper exposure has also been documented among children with a rare genetic condition called Wilson’s disease, which prevents the excretion of excess copper in those with heterozygous alleles [78].

**Table 2:** Examples of increased susceptibility in the human population

Factor	Example	Susceptibility^*a*^	Reference
Examples from *Science and Decisions* 2009 report			
Genetic	Increased copper susceptibility among Wilson’s heterozygotes (~ 1% population).	> 10:1	NRC 2000 [78]
Predisposing exposure	Increased susceptibility among smokers to arsenic-induced lung cancer.	20:1	CDHS 1990 [76]
	Increased susceptibility among smokers to radon-associated lung cancer.	10–20:1	ATSDR 1992 [79]
	Increased susceptibility among low-iodide female smokers to perchlorate-induced thyroid hormone disruption.	20–100:1	Blount et al. 2006 [77]
Preexisting disease	Increased susceptibility among people with hepatitis to liver cancer from aflatoxin.	10–30:1	Wu-Williams et al. 1992 [80]
Physiologic and Pharmacokinetic	Difference in susceptibility to 4-aminobiphenyl (median vs upper 2 percentile) due to physiologic and pharmacokinetic differences (modeled).	> 10:1	Bois et al. 1995 [81]
Overall	Increased heterogeneity in lung and colorectal cancer risk (95th percentile vs median) from age-specific incidence curves.	50:1	Finkel 1995, 2002 [82, 83]
Additional examples identified from peer-reviewed literature			
Age/life stage	Analysis of kinetic data from pharmaceutical studies in populations of adult white, non-white, children, and those with metabolic polymorphisms which found 10-fold variation (99.9% protective) too low for very young.	> 10:1	Renwick and Lazarus 1998 [84]
	Greater susceptibility among elderly populations compared to adults.	> 10:1	Abdel-Megeed 2001 [85], Skowronski et al. 2001 [86]
	Under-protective of children.	> 10:1	Hattis et al. 2002 [87]
	Decreased expression or activity of xenobiotic metabolizing enzymes (e.g., cytochrome p450) in 2nd trimester fetal livers compared to adult livers.	77–12,271:1	Robinson et al. 2020 [88]
Age/life stage and genetic	Not protective of neonates, elderly, or people with polymorphisms.	> 10:1	Dorne et al. 2007 [89]
	Increased TK susceptibility among children/infants and occupational groups with genetic susceptibility.	1 to 30–60:1	OEHHA 2008 [90]
Toxicokinetic	Increased variation in metabolic clearance of trichloroethylene (TCE).	10–100:1	Chiu et al. 2014 [91]
Toxicodynamic	Increased variation in cytotoxic response to specific chemicals in population-based human in vitro models (95th percentile vs median) using 1000 Genomes Project.	1 to 10–100:1	Abdo et al. 2015a [92]

^*a*^ Examples adapted from the NRC *Science and Decisions* 2009 report [1] and peer-reviewed literature. Increased susceptibility determined based on susceptible case to “normal” ratio as listed in Table 4–1 of 2009 NRC report [1]

Additionally, data not included in the NAS report from the scientific literature show that human variability across chemicals often exceeds 10X, including examples such as greater susceptibility to chemical exposures among young infants/children, the elderly, and people with underlying disease or obesity (Table 2). Further evidence supporting wide variability in human responses to chemical exposures comes from demonstrated differences in metabolic capacity and functioning across the lifespan. For example, the expression and activity of several major enzymes (e.g., cytochrome p450s) critical for detoxification of compounds is very limited during early life stages (embryo/fetus/infancy) and increases with adulthood. Several important cytochrome p450 (CYP) enzymes are reduced in the fetus and at birth, only reaching 30–50% of adult levels by 1 year of birth, which can decrease the ability to metabolize toxic chemicals such as benzene, trichlorethylene, and toluene [90]. A recent study which used in silico and molecular-based methods for quantifying the variability in expression or activity of CYPs reported significant differences between adult and fetal livers [88]. Major xenobiotic metabolizing enzymes such as CYP1A1, −1A2, −2B6, −3A4, and -2E1 were expressed 77X, 1528X, 1224X, 216X, and 12,271X lower in 2nd trimester fetal livers versus adult livers, respectively [88]. Thus, deficiencies in metabolic capacity at early points in human life can directly impact toxicokinetics leading to differences in circulating toxicant concentrations and downstream effects. In addition, certain physiological parameters of homeostasis that are specific to age can make individuals more or less susceptible to environmental exposures [93]. For example, the ratio of liver size to body mass is higher in early life, which increases metabolic clearance among children compared to adults [90]. Additionally, young infants (less than three months old) have lower lipid content with respect to adults (reducing their relative retention of lipophilic chemicals) while older infants have higher lipid content with respect to adults (increasing their relative retention of lipophilic chemicals such as polychlorinated biphenyls [PCBs] and dioxins) [90]. Likewise, elderly populations can have greater susceptibility to chemicals such as pharmaceuticals due to decreased metabolic capacity and ability to respond and repair physiological damage with older age [90]. Indeed, researchers have observed increased half-lives of pharmaceuticals by 60% and decreased drug clearance by 50% in elderly populations compared to other adults [94].

Two examples of improvements upon standard practice at the federal level have been demonstrated by the California EPA’s Office of Environmental Health Hazard Assessment (OEHHA). First, a comprehensive review of this literature (i.e., age-dependent toxicokinetic summaries) and in-house physiologically based pharmacokinetic data modeled by OEHHA found an approximately 10-fold difference between children or young infants and adults exposed to several hazardous air pollutants in kinetic variation alone [90]. A point estimate adjustment factor of 10X for TK variability (AF_H-TK_ = 10) multiplied by 3X for tissue response variability (AF_H-TD_ = √10 = 3.16) would translate to an adjustment factor of about 30-fold for intra-species variability (AF_H_ ~ 30) [90]. Second, OEHHA set a reference exposure limit for benzene using a chemical-specific human variability adjustment factor of 60X based on literature showing a wide range of TK variability in response to benzene exposure among Chinese workers, with differences largely due to three genetic polymorphisms that increased susceptibility up to 20-fold [90]. This resulted in a California chronic reference exposure level (REL) that was 70% lower than the US EPA RfC for the same critical effect of decreased peripheral blood cells in chronically-exposed workers [95]. Another chemical-specific example is for trichloroethylene (TCE) where Chiu et al. demonstrated 2-fold to 7-fold variation (upper confidence limit of 31-fold) (depending on metabolic pathway) in the metabolic clearance of TCE and underlying disease risk by accounting for multiple sources of population variability and data streams (both human and animal) [91, 96]. The authors demonstrated up to 31-fold variability in the ratio of metabolic flux through oxidation compared with glutathione conjugation across 17 diverse mouse strains [91].

While there is less quantitative data on TD variability compared to TK variability (in terms of whether the 3X factor for TD variability is sufficient to be protective of susceptible subgroups within the population), data on physiological processes of development show that fetuses and young children are more susceptible to chemical exposures than adults. For example, developing fetuses are more susceptible to the neurodevelopmental effects of heavy metals and the reproductive effects of exposure to endocrine disrupting compounds (e.g., diethylstilbestrol [DES]) [90]. This increased susceptibility occurs because the embryonic/fetal brain undergoes rapid growth and is highly susceptible to chemicals (e.g., heavy metals) that alter cell proliferation during gestation, while hormone disrupting chemicals like DES are hypothesized to disrupt active periods of human reproductive development (e.g., fetal development, adolescent puberty) due to their ability to mimic endogenous hormones and alter pathways critical for growth and differentiation [97]. Children can also be more susceptible to chemicals that cause or exacerbate asthma, as identified in OEHHA’s hot spots report [90]. The utility of cell-based assays has been demonstrated by Abdo et al. through the use of population-based human in vitro models [92, 98]. The authors used 1086 lymphoblastoid cell lines from the 1000 Genomes Project (representing nine populations from five continents) to estimate inter-individual variation in cytotoxic response for 179 chemicals. The cytotoxic response in the 1% most susceptible cells occurred at concentrations at factors of greater than ≤10 (~ 3X) from the median response for about half of the chemicals tested, and up to 100-fold for some chemicals tested [92], further indicating from in vitro studies that the 10-fold default adjustment factor is not reflective of chemical response variability in the human population.

Additionally, the World Health Organization’s International Programme on Chemical Safety (IPCS) found up to a 42-fold range of human variability in response to chemical exposures when human TK and TD was combined probabilistically using data primarily from healthy adults [16]. For this analysis, the IPCS used separate TK and TD data sets compiled by Hattis et al. (each with separate sets of roughly 30 chemicals tested, mostly in controlled studies of pharmaceuticals in healthy human adults) to generate generic human TK and TD probability distributions that can be applied to represent TK or TD variability for chemicals lacking chemical-specific TK or TD variability data [16, 87, 99]. The 42-fold variation was specific to a target population disease incidence of 1% (with 95% confidence to capture the upper end of the distribution of variability across chemicals), while a 14-fold variation was estimated with respect to a 5% target incidence (with 95% confidence). These findings further suggest that (based on human data) a 10-fold factor is insufficient to protect the population from chemical exposures.

### Improved approaches and opportunities for better use of science

Risk assessors and authoritative bodies have recommended the use of probabilistic approaches that account for human variability and adjustment factor distributions [15, 73, 87, 99–102]. As noted, in 2002 Hattis et al. proposed a widely cited probabilistic approach to account for human variability in chemical risk assessment using a distribution of values instead of a point estimate (default adjustment factor) to represent the range of human response to chemical exposures at varying doses [16, 87]. The human variability distribution approach provided a quantitative method to predict the probability of human response to chemical exposure based on a range of TK and TD values, rather than assuming that 10X is sufficient to protect the entire population for all chemicals. This advance also allowed for extrapolation from high to low response rate doses which improved the use of animal data for human toxicity prediction [16, 87]. A probabilistic method that evolved from the Hattis et al. 2002 study has been developed by the IPCS to adjust for population differences in susceptibility to chemical exposures using the generic human variability distributions described above [16, 87, 102]. These probabilistic methods allow for the AF to be represented by a distribution rather than point value, which is useful because it enables estimation of risk instead of an RfD or other similar value that does not represent a quantified risk (this is further discussed in the companion paper on probabilistic approaches to estimating risks for non-cancer health effects by Nielsen et al. in this issue). The IPCS methods and adjustment factor distributions generated by the IPCS using the Hattis TK/TD data sets have since been applied to ~ 600 chemicals by Chiu et al. [101].

#### Probabilistic data to characterize human variability

The method presented by Hattis et al., and further extended by IPCS and Chiu et al., is important for future consideration of human variability in risk assessment for two reasons. First, it makes use of available data (primarily from pharmaceuticals) to develop the concept of human variability as a distribution of susceptibility in the population that varies across chemicals. Second, it provides a method for using human variability distributions to derive probabilistic estimates of non-cancer risk, in place of the traditional RfD. However, the human TK and TD variability distributions derived from datasets compiled by Hattis et al. have several important limitations which can underestimate human variability. Namely, the information is based primarily on data from healthy adults and does not capture the range of endpoints or the full range of factors that influence variability including life stage, chronic conditions, and other intrinsic and extrinsic factors that influence susceptibility. The approach is presented as a proof of concept rather than as a definitive distribution of human variability. While this method was recommended and further advanced by the NRC in 2009 and the IPCS in 2014 [1, 75], both reports emphasized the importance of updating the approach with new data, such as newly-available chemical-specific dose-response data, as they become available.

**Fig. 4:**
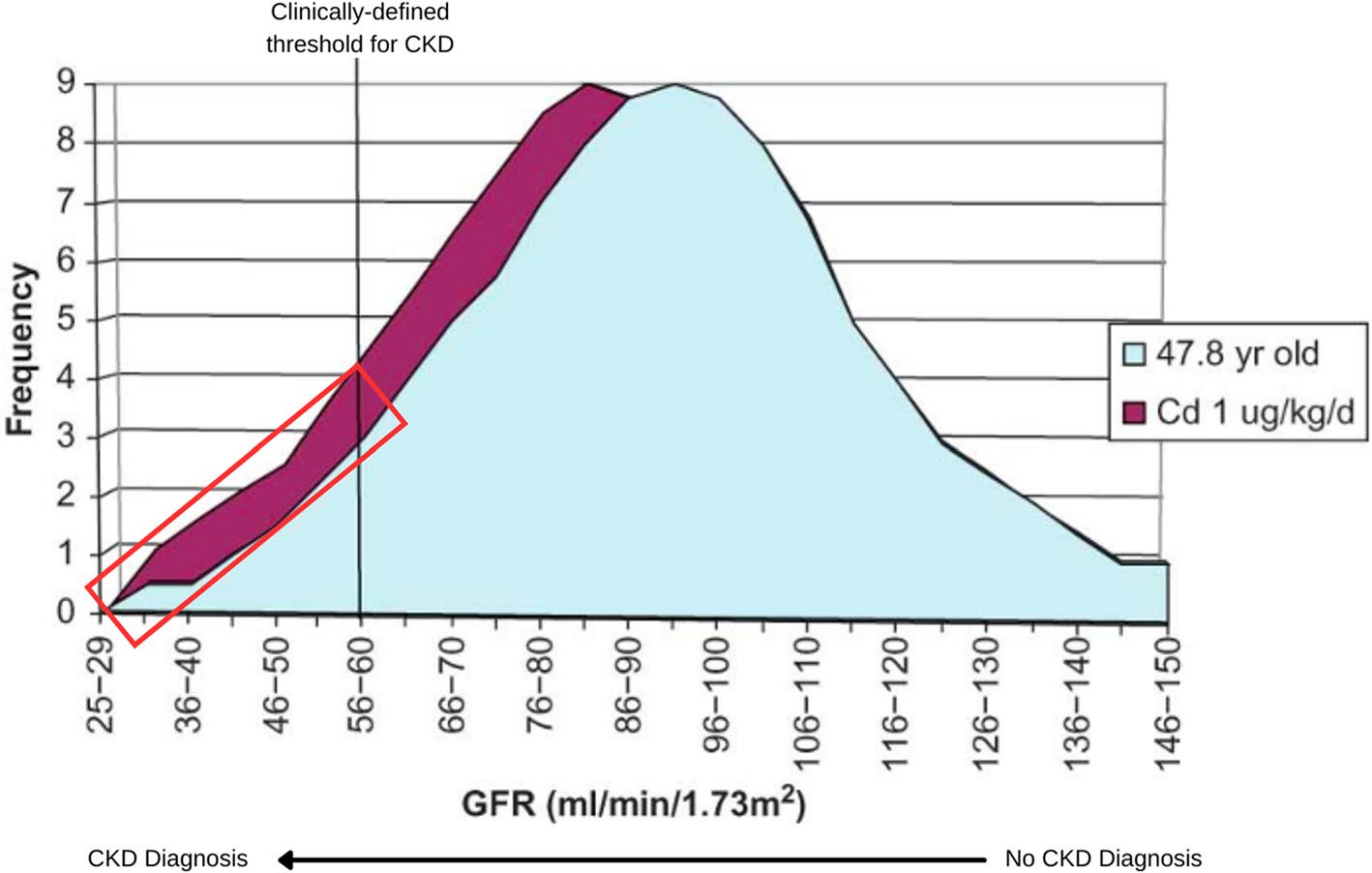
Additive risk of chronic kidney disease (CKD) due to cadmium exposure Cadmium risk assessed in relation to background risk (blue curve) of CKD for a single age group (47.8-year-old women). CKD is diagnosed with a glomerular filtration rate (GFR) of less than 60 ml/min/1.73m2. A reduction in the GFR distribution (red curve) from a chronic cadmium exposure of 1 µg/kg/d increases the population risk of CKD (red box represents increased portion of the population with GFR below the diagnostic threshold) by 3.7%. Reproduced with permission from Ginsberg 2012

Several examples in the literature demonstrate how to integrate multiple emerging data sources and advanced statistical techniques to better approximate human population variability for specific chemicals. An approach which uses population-level data on background disease risk combined with epidemiologic data on chemical exposures that is more representative of endpoint-specific variability in the population has been illustrated by Ginsberg [103]. The authors demonstrated a clinical variability approach to account for underlying chronic condition and disease risk by combining population-level data on age-adjusted chronic kidney disease (CKD) risk and glomerular filtration rate (GFR), a continuous biological measure of kidney function and indicator of age-related decline that is also a clinical predictor of CKD. The combination of the disease risk distribution with data on the effect of cadmium (Cd) exposure on GFR enabled the estimation of the impact of Cd on CKD [103]. The method was able to capture the age-dependent baseline (underlying) population distribution (normally) and then quantify the expected shift in the baseline distribution (for each age group) based on the expected association between Cd exposure and GFR, and the relationship between GFR and risk of CKD (Fig. 4). The analysis found that a 0.1 μg/kg/day intake of Cd leads to three additional cases of CKD per 1000 adults exposed due to the link with GFR. The advantage of this method is that it can interface the breadth of human variability for a key underlying predictor of disease risk with the dose response for chemical effect on that disease risk factor, but it requires the adverse effect of the chemical exposure (Cd) and the clinical outcome (CKD) be defined and linked by the same continuous measure (GFR) and is thus highly dependent on data availability.

#### Epidemiologic data to characterize human variability

Epidemiologic studies also have the potential to capture human variability and susceptibility if the study population is representative of the general population and/or includes susceptible subgroups. For example, data from the National Health and Nutrition Examination Survey (NHANES) and large cohort studies, such as the Nurses’ Health Study, Framingham Heart Study, and the Environmental influences on Child Health Outcomes (ECHO) Program, could be leveraged for use in cumulative risk assessment if the populations examined include a reasonably representative sample of the population (for human variability estimation on a specific endpoint) or a susceptible subpopulation (such as an age-specific or racial/ethnic subgroup). New methods have also emerged from social epidemiology for assessing the combined effect of exposure to chemical and non-chemical stressors (e.g., psychosocial stress, air pollution, and asthma; and studies investigating allostatic load with exposure to lead), with advanced statistical techniques that include latent variable modeling and structural equation modeling which facilitate multilevel modeling of complex data [104]. These epidemiologic analyses can be used to quantify variability in susceptible subgroups if the populations examined include biologically susceptible and/or socially disadvantaged subgroups. Additionally, susceptibility factors can be estimated from survey research (e.g., US Census “American Community Survey” [105], the Behavioral Risk Factor Surveillance System (BRFSS), special studies) [106], and geographic information system (GIS) techniques and principal component analyses have been used to develop indices and characterize the geospatial location or cumulative exposure of susceptible and highly exposed groups (e.g., EPA’s EJScreen [64, 107], CalEnviroScreen [108, 109], MI-Environment [for heat, but social factors are combined [110]], Community Approaches to Promoting Healthy Environments (CAPHE) [for air pollution in Detroit [111]], and others [2, 112]).

#### Alternative test methods to characterize human variability

The use of new approach methods (NAMs), including high-throughput screening (HTS) technologies to evaluate human health risks from chemical exposures, is gaining momentum globally, due to a focus on decreasing use of in vivo versus animal-free toxicity testing, the relatively low cost of these methods, and the ability to assess multiple experimental conditions and endpoints in a rapid manner [113, 114]. These large-scale, diverse efforts include the development of 1) relevant in vitro tests for toxicity screening; 2) public “big data” repositories relevant for environmental health and toxicology; 3) computational models that use in vitro test results to predict biological response to chemical exposures; and 4) in silico frameworks which guide in vitro to in vivo extrapolation analyses. For example, the National Toxicology Program-sponsored Developmental NeuroToxicity Data Integration and Visualization Enabling Resource (DNT-DIVER) project has enabled the development and coordination of diverse in vitro models to identify environmental chemicals with potential to cause developmental neurotoxicity [115]. The US EPA ToxCast repository includes data for approximately 1800 chemicals (though mostly pharmaceuticals) in over 700 unique endpoints, broadly covering a diverse range of bioactivities in high-throughput assays [116]. Computational tools such as EPA’s Virtual Embryo (v-Embryo™) project which integrate in vivo, in vitro and in silico data, are used to simulate critical aspects of embryonic/fetal development (e.g., vascular development [117], blood-brain barrier formation [118]) and predict chemical toxicity in silico during sensitive points in gestation.

While relatively unexplored, NAMs may be utilized to predict human variability in chemical response. As discussed above, research by Abdo et al. using diverse human lymphoblastoid cell lines of worldwide representation demonstrated a wide range of variability in cytotoxic response to 179 specific chemicals [92] and suggested that comparisons between the 99th percentile and the median may be useful as an AF for human TD variability in chemical risk assessment, illustrating an approach for using high-throughput in vitro data to derive a human toxicodynamic variability distribution. In a similar manner, Burnett et al. applied a panel of 43 unique human stem cell lines to test for the variability in cardiotoxicity response of 134 chemicals, evaluating nine different phenotypes critical for cardiomyocyte performance, and discovered chemical-specific variability in potency and degree of population variability, with higher relative potency associated with higher population variability [119]. As these types of studies expand to other models and endpoints, including those of higher relevancy to human physiology, exposure and response, these in vitro approaches to quickly assess large numbers of chemicals in panels of diverse human cell lines may serve as an improved proxy for human toxicodynamic variability as compared to standard default AFs. Interestingly, observations of variability in chemical response in select NAMs (e.g., cell lines [119], zebrafish [120]) are already being leveraged to pinpoint specific genotypes linked with susceptibility to particular environmental chemicals and pharmaceuticals due to the ease and value in performing these studies as compared to large-scale population-based human studies.

Investigations leveraging genetically diverse rodents (e.g., the Hybrid Mouse Diversity Panel, the Collaborative Cross (CC), or the Diversity Outbred (DO) models [93, 121]) have also provided tremendous insight in terms of toxicological response and methods for quantifying variability. For example, as discussed above, Chiu et al.’s Bayesian statistical approach to estimate the range of variability in response to TCE in genetically diverse strains of mice [91] may be applied in models assessing human response variability. Furthermore, as scientists establish more representative models (e.g., tissue/organ bioengineered models) of human development for toxicological investigations as well as more sophisticated statistical approaches to model these data more accurately [122], we expect that NAMs will prove to be more useful at predicting the genetic component of human variability.

More research is needed to define the ability of in vitro and in silico models in predicting human toxicity and variability in response. Some current limitations of in vitro studies include limited consideration of metabolism and over-emphasis on acute toxicity, leaving gaps in knowledge regarding more/less reactive chemical metabolites and the effects of chronic exposures, areas that have yet to be addressed. Another limitation of emerging probabilistic methods is that while they may better capture variability with respect to biological susceptibility, they do not capture external social factors, like exposure to non-chemical stressors related to, for example, systemic racism and lower socioeconomic status. Due to these and other limitations, the EPA Children’s Health Protection Advisory Committee emphasized in a recent report to EPA that its use of mechanistic and high throughout data should be used to strengthen evidence and upgrade an evaluation, but not to weaken or downgrade evaluations [123].

### A path forward: recommendations to address evidence and policy needs

In summary, new data and current science should be used to improve accounting for human variability and susceptibility in hazard and risk assessment. We recommend three overlapping approaches for how to implement the recommendations below, including the use of 1) computational tools and methods such as probabilistic approaches to quantify generic probability distributions; 2) data from genetically diverse in vivo and in vitro animal models and human observational studies or clinical trials to inform human variability; and 3) epidemiologic data and cumulative impact assessment tools to capture extrinsic factors for characterizing human variability. Further, we recommend the following improvements to incorporate human variability into chemical risk assessment with more specific recommendations outlined below:

- Increase the default human variability factor to better account for intra-species variability
- Incorporate additional categories into human intra-species variability adjustment factors
- Use the clinical variability approach when sufficient data are available
- Apply probabilistic approaches that integrate multiple heterogeneous data sources

The first two recommendations are both related to the current default 10X factor approach that uses a single value adjustment factor to account for human variability, as typically applied in current US EPA risk assessments for non-cancer effects (i.e., reference values). In contrast to this point estimate approach, probabilistic approaches such as those captured by the second two recommendations are preferred to advance chemical risk assessment going forward. Additionally, while there is ample data to improve how we characterize human variability, there are still limitations in the available data, as noted above. As such, we also emphasize that these approaches, including mechanistic and high throughput data (such as the use of sensitive study designs that capture early life exposure and/or use diverse human cell and/or outbred animal models), should be used as a minimum level default to upgrade (but not downgrade) consideration of these factors.

#### Recommendations related to the current default 10X factor for human variability

As discussed, OEHHA’s alternate default adjustment factor (which increased the current default factor from 10X to 30X) is based on evidence of increased TK susceptibility to benzo [a] pyrene, TCE, and other air toxicants among susceptible age groups (i.e., young infants and children) [90]. Additionally, as noted, an approach used by the IPCS in 2014 demonstrated that an adjustment factor of about 42X is needed to address human variability for a target population incidence of 1% (i.e., a value protective of the 99th percentile) with 95% confidence, based on human variability data sets reported by Hattis et al. in 2002 [16, 87, 99]. Table 3 summarizes existing approaches to chemical risk assessment and their limitations (Table 3).

**Table 3:** Default and probabilistic approaches to intra-species adjustment for human variability and consideration of susceptible subgroups

Agency	TK Subfactor	TD Subfactor	Total Intra-species AF	Type	Subgroup Consideration
US EPA	√10 ≈ 3	√10 ≈ 3	10-fold	Default	Does not explicitly account for sensitive subgroups.
Cal EPA (OEHHA)	10	√10 ≈ 3	30-fold	Default	Children/infants and genetic TK susceptibility (though does not cover fetal period nor all identified variabilities).
IPCS/WHO	2–4.5^*a*^	2.5–10^*a*^	3.5–42-fold^*a*^	Probabilistic	Does not explicitly account for sensitive subgroups; data based primarily on studies in healthy adults.

Intra-species adjustment (sub) factors representing human metabolism (TK) and tissue response (TD) TK/TD variability

^*a*^ IPCS ranges include median and 95th percentile estimates for target population incidence ranging from 1 to 10% [16]

##### Increase the default human variability factor to better account for intra-species variability

Due to the significant body of scientific evidence supporting the need for greater public health protection among susceptible and highly exposed populations, we recommend that as a first step, the default adjustment factor for intra-species variability used by the US EPA should be increased to a minimum of 42X, unless there are robust chemical-specific data to the contrary. This is supported by the 42X IPCS estimate of human variability, which relied on high-quality TK and TD data, with the limitation of primarily focusing on healthy adults [16, 99]. It is also critical to account for human variability in cancer dose-response analysis, a recommendation of the NAS in *Science and Decisions* [1, 124, 125]. The NAS recommended a default assumption of a 25-fold difference in cancer risk between the 95th percentile and the median human response; current cancer dose-response methods incorporate only the estimated response at the median of the population [1, 125].

##### Incorporate additional categories into human intra-species variability adjustment factors

Based on the scientific literature [6, 15, 16] and authoritative bodies [1, 5, 12, 13, 126], such as the National Research Council and the US EPA, we recommend the following categories, though not exhaustive, should be included in human variability adjustment factors to improve their scientific basis.

**Separate adjustment factor to address age-related susceptibility**

We further recommend adoption of a separate adjustment factor for age/life-stage differences, which is supported by the IPCS and OEHHA analyses described above [16, 90]. Although some overlap exists between the 42X recommendation above and the 30X default factor adopted by OEHHA, the IPCS analysis found 42X human variability considering only (to a large extent) data from healthy adults and very little data for other life stages [16], while OEHHA’s adoption of an increased default adjustment factor of 30X was primarily based on age-specific differences in chemical metabolism (between children and adults) [90]. Thus, the 42X recommendation above reflects differences among adults and not differences across age/life stage of development, while OEHHA’s 30X factor does not account for human variability from every susceptible life stage (i.e., in utero development, pregnancy, etc.), further indicating that separating these adjustment factors is warranted. A separate adjustment factor for age-related susceptibility has already been required by Congress for food-use pesticides where an additional factor, usually 3X or 10X, called the Food Quality Protection Act (FQPA) safety factor, is incorporated into risk assessments for addressing additional susceptibility for pregnant women and children exposed to pesticides [127]*.* This approach is supported by evidence demonstrating that the current default approach does not protect the human population across life stages of development when considering age-specific differences, such as decreased metabolic clearance of pharmaceutical chemicals among developing fetuses and young infants compared to adults [128] (with additional examples outlined in Table 2 above). Moreover, a separate category for life-stage adjustment could better incorporate TD differences that the default OEHHA 30X factor, which is based on TK differences, does not address. Finally, we also recommend harmonization and adjustment for age-related susceptibilities in both cancer and non-cancer risk assessment. The US EPA Cancer Guidelines currently recommend an additional adjustment factor where children may be exposed to mutagenic carcinogens [129]. However, it’s important to note that there is no early life adjustment factor for carcinogens with other or unknown modes of action [129].

**Separate adjustment factor to address multiple chemical and non-chemical stressors**

We recommend development of a separate default extrinsic variability factor (in addition to the 42X and age-related factors above) that would account for exposure to multiple chemical and non-chemical stressors [66, 67]. This factor could account for human variability in susceptible subgroups, potentially leveraging data on allostatic load and ubiquitous population-level chemical exposures like lead [130, 131]. If the populations studied are representative of the general population and/or include susceptible subgroups, large epidemiologic cohort studies, such as the Nurses’ Health Study, the Framingham Heart Study, and the ECHO Program, could be mined for endpoint-specific variability in susceptible subgroups, which could be used to inform both chemical-specific and generic estimates of response variability in susceptible subgroups. New methodologies for combining chemicals in a potency-based, data-driven, or hybrid mixture modeling framework should also be prioritized to advance consideration of susceptibility in human health risk assessment, while risk assessments should routinely include explicit descriptions of susceptible subgroups and analysis of current data on multiple sources of variability within those groups. If data suggest, for example, interactive effects associated with low birth weight (LBW) are much stronger among Black women compared to other groups, an additional adjustment factor (one that is separate from the 30X default factor recommended above) should be applied (e.g., when LBW is an effect of the chemical being assessed). Ultimately, we recommend science-based tools that address human variability and susceptibility in cumulative risk assessment frameworks. Examples include health impact assessments, public health tracking and biomonitoring like NHANES, geospatial tools like CalEnviroScreen, and the Environmental Justice Screening Method (EJSM) [4, 5, 18, 132, 133]. These tools can be used to identify susceptible populations for prioritization in risk assessment and human variability estimation, which may be particularly appropriate for addressing requirements under multiple statutes, including ongoing risk evaluations under TSCA [134], maximum contaminant levels (MCLs) and the Drinking Water Contaminant Candidate List (CCL) under the Safe Drinking Water Act (SDWA) [135, 136], and risk determinations for air toxics under the Clean Air Act (CAA) [137].

#### Recommendations to incorporate new approaches to account for human variability in EPA risk assessment research and practice (replacing the approach of applying a single-value adjustment factor)

##### Use clinical vulnerability approach to address underlying chronic condition or disease risk

When sufficient data are available, estimating disease risk in a population that is representative of the general population and/or susceptible subgroups can better account for variability in a specific response. The method of Ginsberg 2012 [103] can help address underlying susceptibility in baseline chronic conditions and disease risk, and the cadmium-GFR example could be extended to susceptible populations (e.g., examining adjusted GFR among pregnant people and/or racial/ethnic or low SES subgroups) [103, 138]. Options for applying this method to cardiovascular disease could include examining the increased effect of mercury on the fatty streak of the carotid artery (using data from carotid artery imaging) [138], as has been demonstrated for mercury and other cardiovascular endpoints [139]. Similar applications could examine the additive effect of air pollution, lead, or dioxin on heart rate or blood pressure in susceptible populations; neurotoxic effects; or TCE-induced autoimmunity [138].

##### Apply probabilistic approaches that integrate multiple heterogeneous data sources

We recommend that agencies adopt probabilistic approaches that integrate multiple heterogeneous data sources (human, animal, in vitro*,* in silico) to quantify overall and category-specific human population variability in chemical risk assessment. The incorporation of new data could serve to update generic default probability distributions for TK and TD variability that were established by the IPCS in 2014 [99] as described by Axelrad et al. 2019 [16]. For example, the generic probability distribution created from the Hattis et al. distributions on pharmaceuticals could be updated with chemical-specific TK information on environmental chemicals as the data become available. Integrating diverse data sets could also improve the use of probabilistic distributions for specific sources of variability and inform adjustment factors used in chemical risk assessment [16], advancing specific areas that are currently lacking. For example, in vitro data such as the approach used in the 1000 Genomes study by Abdo et al. may be the most efficient way forward to expand (but not decrease) health protections by more fully characterizing the genetic component of human TD variability, with cell types other than lymphoblastoid cells and assays that reflect a broader range of endpoints than cytotoxicity, while heterogeneous animal strain populations can inform efforts to better characterize TK variability in the human population. Emerging high-throughput and data-rich models (i.e., heterogeneous human in vitro and animal in vivo) have the potential to rapidly generate a large amount of data that is relevant for human health chemical risk assessment. Thus, combining diverse data sources and data types (human epidemiological, human controlled, animal and human in vitro, etc.) would more comprehensively capture total population genetic and non-genetic variability.

We therefore recommend integrating these diverse updated data sources (including controlled and epidemiologic human studies, animal models, and expanded sets of in vitro cell lines/endpoints) to update overall (i.e., genetic and non-genetic) and/or category-specific (e.g., susceptible life stages, low SES subgroups) human variability, as described by Axelrad et al. [16]. Moreover, we recommend the EPA develop a Bayesian framework for updating human variability distributions incorporating different types of data that are informative about different components of variability (TK vs. TD, genetic vs. non-genetic, etc.) as a way to integrate the existing Hattis et al. approach with emerging data from epidemiologic, in vitro, and genetically diverse animal data sets as new data become available, as exemplified by Chiu et al. and others [101]. Updated methods should make use of the best available science, but these methods should never be employed to weaken risk estimate or exposure limits. Similar recommendations have been made in the past for combining multiple data streams into a holistic framework that allows for the examination of intrinsic and extrinsic factors in combination [3, 4, 140] and that provides risk assessors with a tool chest of options for addressing variability in chemical risk assessment that is more protective of susceptible subgroups [93, 100].

##### Use evidence-based systematic review for adjustment factor refinement

Previous publications assessing the default factor of 10X have provided useful perspectives on the sufficiency of a 10-fold adjustment as well as methods to evaluate the extent of human variability. To ensure greater transparency in assessments of human variability, we recommend the formal use of the University of California San Francisco’s (UCSF’s) Navigation Guide systematic review framework [141], or another which meets similar standards such as the National Toxicology Program’s Office of Health Assessment and Translation (OHAT) approach [142, 143], in human health chemical risk assessment to better incorporate current data on variability and susceptibility in a methodical, standardized, and reproducible framework. For example, using a pre-published protocol, a systematic review could be performed on the body of epidemiologic literature that has emerged from seven research projects funded by the US EPA over the past decade to examine the combined effects of environmental chemical exposures (e.g., air pollution, lead, mercury) and non-chemical stressors (i.e., psychosocial stressors) on various health endpoints (e.g., asthma and neurological outcomes) using diverse modeling techniques (e.g., latent variable and structural equation modeling) [104]. This systematic approach could also be used to identify data sources for probabilistic modeling, which is highly dependent on the quality of data-rich sources.

In summary, current data (such as the data presented in this paper from OEHHA, the IPCS, and the scientific literature) show that the approaches used to incorporate human variability in hazard and risk assessment need to be updated to reflect current science. The US EPA should improve its risk assessment practice by adopting a higher and more accurate default point estimate adjustment factor for inter-human variability and potential separate adjustment factors for age-related and external sources of variability. The agency should also advance risk assessment research and practice by implementing probabilistic approaches that integrate information across multiple complex data sets. Updated approaches that integrate multiple data sources would improve estimates of human variability and provide more adequate protection for susceptible subgroups, such as pregnant people, developing fetuses/neonates, children/adolescents, low-wealth populations, and those burdened by additional occupational and/or environmental exposures. Protective approaches for susceptible groups (e.g.*,* pregnant people, lactating people, people with disabilities, and other susceptible subgroups) would ultimately achieve overall population benefits for all.

## Data Availability

Data sharing is not applicable to this article as no datasets were generated or analyzed during the current study.

## Abbreviations

AF_A_: Animal-To-Human Adjustment Factor
AF_H_: Human Variability Adjustment Factor
AF_H-TD_: Intra-Species Human Variability Adjustment Subfactor for Variation in Target Tissue Response
AF_H-TK_: Intra-Species Human Variability Adjustment Subfactor for Variation in Kinetic Chemical Metabolism
AFs: Adjustment Factors
BMDL: Benchmark Dose
BRFSS: Behavioral Risk Factor Surveillance System
CAA: Clean Air Act
CAPHE: Community Approaches to Promoting Healthy Environments
CC: Collaborative Cross
CCL: Drinking Water Contaminant Candidate List
Cd: Cadmium
CKD: Chronic Kidney Disease
CSAF: Chemical-Specific Adjustment Factor
CYP: Cytochrome p450
DDAF: Data-Derived Extrapolation Factor
DES: Diethylstilbestrol
DNT-DIVER: Developmental Neurotoxicity Data Integration and Visualization Enabling Resource
DO: Diversity Outbred
ECHO: Environmental ﻿Influences ﻿on Child Health Outcomes
EDCs: Endocrine Disrupting Chemicals
EJSM: Environmental Justice Screening Method
EPA: Environmental Protection Agency
FQPA: Food Quality Protection Act
GFR: Glomerular Filtration Rate
GIS: Geographic Information System
HTS: High-Throughput Screening
IPCS: International Programme on Chemical Safety
LBW: Low Birth Weight
LOAEL: Lowest Observed Adverse Effect Level
MCLs: Maximum Contaminant Levels
NAAQS: National Ambient Air Quality Standards
NAMs: New Approach Methods
NAS: National Academy of Sciences
NEJAC: National Environmental Justice Advisory Council
NHANES: National Health and Nutrition Examination Survey
OEHHA: Office of Environmental Health Hazard Assessment
OHAT: Office of Health Assessment and Translation
POD: Point of Departure
REL: Reference Exposure Level
RfC: Reference Concentration
RfD: Reference Dose
SDWA: Safe Drinking Water Act
SES: Socioeconomic Status
TCE: Trichloroethylene
TD: Toxicodynamic
TK: Toxicokinetic
TSCA: Toxic Substances Control Act
UCSF: University Of California San Francisco
US: United States
v-Embryo: Virtual Embryo
